# The Effect of Layer Thicknesses in Hybrid Titanium–Carbon Laminates on Low-Velocity Impact Response

**DOI:** 10.3390/ma13010103

**Published:** 2019-12-24

**Authors:** Patryk Jakubczak, Jarosław Bieniaś, Magda Droździel, Piotr Podolak, Aleksandra Harmasz

**Affiliations:** Department of Materials Engineering, Faculty of Mechanical Engineering, Lublin University of Technology, 20-618 Lublin, Poland; j.bienias@pollub.pl (J.B.); p.podolak.of@gmail.com (P.P.); aleksandra_harmasz@wp.pl (A.H.)

**Keywords:** FML, carbon composite, impact, metal volume fraction, low-velocity impact

## Abstract

The purpose of the work was the effect of metal volume fraction of fiber metal laminates on damage after dynamic loads based upon the example of innovative hybrid titanium–carbon composite laminates. The subject of the study was metal–fiber hybrid titanium–carbon composite laminates. Four types of hybrid titanium–carbon laminates were designed with various metal volume fraction coefficient but constant thickness. Based on the results, it can be stated that changes in the metal volume fraction coefficient in the range of 0.375–0.6 in constant thickness titanium–carbon composite laminates do not significantly affect their resistance to impacts in the energy range of 5–45 J. It was concluded that there were no significant differences in maximum force values, total contact time, and damage range. Some tendency towards a reduction in the energy accumulation capacity was observed with an increase in thickness of the metal part in relation to the total thickness of the laminate, especially in the lower impact energy range. This can result in the lower bending stiffness of laminates with lower metal content and potential elastic strain of the composite part before the initiation of the fiber damage process.

## 1. Introduction

Fiber metal laminates (FML) are a group of hybrid materials composed of alternating layers of metal and polymer-fiber composites [1]. They are characterized by their favorable strength properties, notably particularly high fatigue resistance [2], superior impact resistance [3,4], corrosion resistance [5], and fire resistance [1]. Due to the said properties, but also because of their relatively high production costs, they have found their application mainly in the aviation industry [6].

The continuous development of this group of materials, including the utilization of new metal alloys (titanium alloys [4], aluminim–lithium alloys [1], magnesium [7]) and composites (with carbon fibers, 3D fabrics, thin layers [3,8]), as well as the search for alternative production methods that would reduce costs (semi-automatic and automatic lamination systems) gives the FML laminates significant research and implementation potential.

One of the more prospective materials among next-generation FMLs is the laminate based on titanium and glass or carbon fibers. The use of titanium increases laminate stiffness in comparison to laminates based on aluminim alloys and significantly improves corrosion resistance, i.e., in aggressive environments [9,10,11]. An improvement in the impact behavior of an FML obtained by using new types of metal alloys can be beneficial even if the costs are higher in comparison to the classic solutions (such as aluminum-based FML). This phenomenon is caused by the extra advantages of the new type of FML, such as resistance to perforation and improved energy absorption possibilities [4]. However, few studies address the issue of impact resistance of titanium-composite laminates [9,12,13,14]. Reiner et al. [13] tested a titanium-based FML but failed to address the complex nature of impact phenomena and damage in titanium-based FMLs. Li et al. [9] tested the impact behavior of hybrid titanium–carbon laminates. However, only two criteria (failure modes and energy absorption) were applied to measure the influence of titanium in fiber metal laminates on impact. Li et al. [12] investigated the influence of fiber type on the failure of a hybrid titanium-based FML. Bernhardt et al. [15] characterized the impact response of hybrid titanium–carbon laminates and suggested that the ductility of titanium causes buckling by yielding, whereas the brittle adjacent composite plies lead to fracture. Nakatani et al. [14] concluded that the impact behavior of titanium-based laminates was dominated by fracture in the titanium layer. However, this concerned laminates with glass fibers (metal dominant failure mechanism). All published aspects of impact resistance of titanium-based laminates involve damage or damage criteria, while the lay-up organization or diverse structure of FMLs are not considered. Most recent articles are focused on the standardized structure of FMLs (such as 2/1 or 3/2).

The second element of critical significance for impact resistance is laminate structure. Due to the almost unlimited possibilities of designing the structure of FML laminates, e.g., by using different thicknesses of individual layers, different numbers of metal and composite layers, or using different fiber arrangements [16,17], it is possible to design their properties to match specific requirements. One of the basic parameters describing the structure of FML laminates is their metal volume fraction (MVF), among others [1]. This parameter determines the volumetric ratio of metal in relation to the total volume of the laminate. The MVF factor is used, among other purposes, to predict strength properties (in-plane) for any designed laminate, based on knowledge of the properties of its individual components [1]. On the other hand, one of the most important out-of-plane properties of laminates is their resistance to dynamic loads (impact). Due to the differences in energy absorption and elastic-plastic degradation mechanisms of metal and brittle polymer–fiber composites in response to impact, the use of different FML construction may affect their impact behavior. Zhu et al. [18] demonstrated that there is no clear relationship between MVF and impact resistance. However, the thickness of the laminate is of great importance [19,20,21]. One of the unique features of metal–fiber laminates, important for their impact resistance, is the fact that, within the same overall laminate thickness, it is possible to design laminates with significantly different numbers of layers, thicknesses, orientations, etc., while using the same materials. Because of that, impact behavior evaluations of varying MVF for constant laminate thicknesses were made. According to Wu et al. [22], the performance optimization of layered structures should be carried out by manipulating strain delocalization to a greater extent through further enhancing interfacial adhesion and accelerating the stable propagation of the crack via the thinning of brittle layers. The authors concluded that the thinning of the brittle constituent layer may influence the strain and ultimately affect the mechanical properties. Morinière et al. [23] focused on the ply-angle orientation of GLARE laminates and studied energy distribution in individual layers. The authors concluded that the angle variation of the composite plies had a considerable impact on the impact limit, reaching maximum values when fibers were along the diagonals of the plate. However, the vast majority of impact energy was absorbed through metal, as glass fibers reached rupture early in the damage scenario. An FML concept could provide an impact resistance 70% higher than for the reference at the same weight. Morinière et al. [23] also evaluated the role of the material constituents in impact energy absorption. The authors presented the role of aluminum layers in GLARE. According to the authors, 96% of impact energy was absorbed by aluminum layers: 74% by the impacted aluminum layer and 14% by the aluminum layer farthest from the impacted side. The remaining 12% was due to composite degradation, including delamination initiation and propagation. Based on this, it can be expected that the thickness ratio of components (metal and composite) in FMLs can be a critical factor for energy absorption during impact phenomena, especially in FMLs where the metal plays the dominant role in failure mode (GLARE, titanium/glass laminates). The glass-fiber prepreg layers can withstand considerable deformation and their high membrane stiffness effectively redistributes impact load in the plate’s plane [23], which is the stress state base of FMLs under impact load. However, when the carbon fibers are part of an FML, the composite becomes more significant because of the increase in the fiber dominant failure mode. Morinière et al. [24] evaluated, i.e., aluminum thickness and plate dimensions. As the authors concluded, no significant difference was observed for 0.3 and 0.4 mm thick aluminum. Moreover, the effect of residual curing stresses in FMLs was lower than 1.5%. Based on this, it can be stated that the number of layers with different thermal expansion coefficients should not be an important side effect for impact resistance and stress distribution when the metal volume fraction will be varied. Bikakis et al. [25] concluded that MVF can be used as a qualitative design parameter in order to alter the permanent dent depth and the absorbed impact energy of GLARE plates with equal thickness in a consistent manner, provided that the basic lay-up and number of aluminum and prepreg layers remain unchanged.

Two major factors that can affect the impact resistance of FML materials are a new metal alloy (such as titanium) and the structure and makeup of the FML. Given the literature data dominated by GLARE laminates, it is worth noting that the completely new type of FMLs featuring titanium and carbon fibers is yet to be compared in terms of the influence of the thickness variation of individual layers on impact response. Therefore, the authors of the work have evaluated the laminates with various metal volume fraction under low-velocity impact. The main aim was to identify the global impact response of laminates based on the example of innovative hybrid titanium–carbon composite laminates (HTCL) and numerous impact resistance evaluation criteria.

## 2. Materials and Methods

The subject of the study was the metal–fiber hybrid titanium–carbon composite laminates (HTCL). GRADE2 titanium sheets and high-strength UD carbon-epoxy prepreg (Hexcel, Stanford, CT, USA) were used. Four types of HTCL laminates were designed, and are as follows:

- HTCL type A: Ti/0/0/90/90/0/0/Ti/0/0/90/90/0/0/Ti (total thickness 2.5 mm);
- HTCL type B: Ti/0/0/90/90/0/90/90/0/90/90/0/0/Ti (total thickness 2.5 mm);
- HTCL type C: Ti/0/90/0/Ti/0/90/90/0/Ti/0/90/0/Ti (total thickness 2.5 mm);
- HTCL type D: Ti/0/0/90/90/Ti/90/90/0/0/Ti (total thickness 2.5 mm).

**Figure 1:**
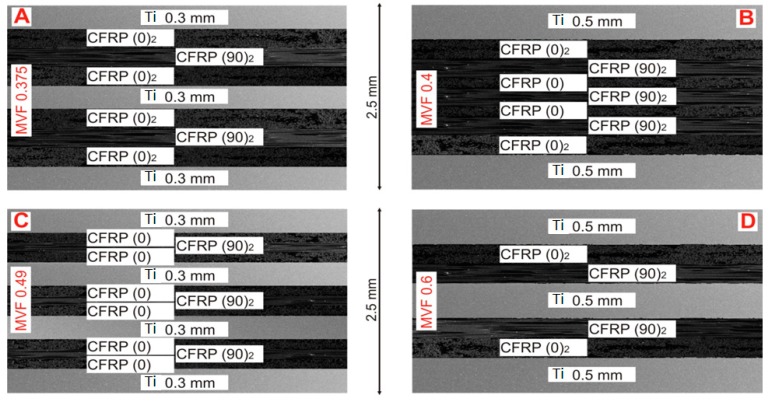
Mesoscale view of hybrid titanium–carbon composite laminates (HTCL) types (**A**–**D**) with constant total thickness and different metal volume fraction.

The above design of HTCL compositions result from the objective of the work, where constant thickness is necessary to obtain the appropriate influence of metal volume fraction in an FML on impact response. The global impact response was compared to HTCL with a constant fiber arrangement (0/90) based on detailed and differentiated assessment criteria. The samples are presented in Figure 1.

The total thickness (*t_lam_*) of each laminate was constant (2.5 mm). The different metal volume fractions were designed by using various titanium numbers and thicknesses, as well as varying numbers of composite layers with the thickness of a single layer of 0.125 mm (Table 1).

The metal volume fraction is defined as the ratio of the sum of the thicknesses of the individual titanium layers and the total thickness of the laminate, see Equation (1) [26].

(1) $$MVF=\frac{\sum_{1}^{p}t_{Al}}{t_{lam}}$$

where:

*t_Al_* = thickness of each separate titanium layer;
*t_lam_* = total thickness of laminate;
*p* = number of titanium layers.

The metal volume fraction as a feature of HTCL was used for impact response comparison and it was selected as a parameter, which can describe the variation of laminate composition in the range of their constant total thickness. Because of a large number and variety of assessment criteria, sensitivity to the impact behavior of HTCL with various compositions was demonstrated. The MVF coefficient was selected because it provides the most global description of FML composition [18,19,20,27,28]. In fact, it should be remembered that the MVF contains the microstructural properties of FML, which are e.g., different interface density, single-layer thickness, thickness ratio, and the stress state in laminate due to the transverse load transfer paths.

The HTCLs were produced in the Department of Materials Engineering at Lublin University of Technology by the autoclave method (Scholz Maschinenbau, Coesfeld, Germany). The cure cycle was carried out at a heating rate of 2 °C/min up to 135 °C and continued at this temperature for 2 h. The pressure and the vacuum used were 0.4 and 0.080 MPa, respectively, through all curing process. The FML panels were cut into samples of 100 mm × 150 mm. Following impact, the laminates were evaluated for damage by means of non-destructive methods [4,29]. Low-velocity impact tests were carried out according to the ASTM D7136 standard [30] using a drop-weight impact tester (Instron Dynatup 9340, Instron, Norwood, Massachusetts, USA). The test was conducted for energy levels 5, 15, 30, and 45 J. More details regarding the experimental data and test stand are presented in other work [4]. Table 2 presents the impact parameters.

The impact force, displacement, energy absorption, bending stiffness, coefficient of kinetic energy, and finally damage by using NDT were analyzed after impact and compared between laminates with various MVF coefficient values. The force was measured using piezoelectric force sensors (Kistler, Winterthur, Switzerland), displacement using photocells, and energy absorption by Equations (2)–(5). The non-destructive tests were performed using a through-transmission phased array immersion system [29].

## 3. Results and Discussion

### 3.1. The Force-Time Curves

**Figure 2:**
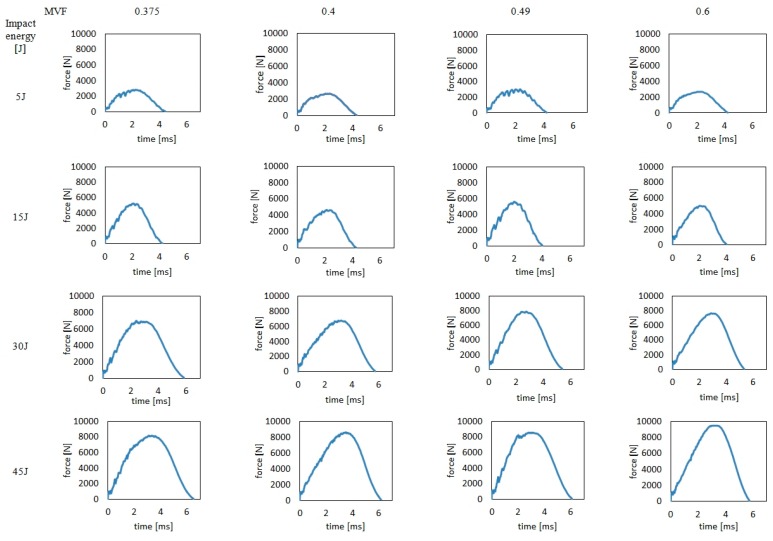
The force–time curves of HTCL with various metal volume fraction (MVF) after low-velocity impact.

The force–time (f–t) curves for hybrid titanium–carbon composite laminates with various metal volume fraction coefficients are found in Figure 2.

The force-time curves of HTCL laminates with different metal volume fraction, recorded during the experiments were characterized by the stage of force increased force-time up to the maximum force (*P*_m_) and the subsequent decrease in force [31]. Regardless of the impact energy, the f-t curves are characterized by an almost symmetrical shape relative to an axis parallel to the force axis, intersecting the time axis for the *P*_m_ value of the force, which indicates the absence of laminate perforation in all of the examined cases [31]. Depending on the impact energy, we recorded different *P*_m_ values and total indenter-laminate contact times. In the energy range of 5 and 15 J, the force values reached about 3000 and 5000 N, respectively, while the total time of contact of the indenter with the laminate was similar and was about 4 ms. In the 30 and 45 J energy range, the forces reached approximately 7500 and 9000 N, respectively, with a total indenter penetration time and withdrawal phase of approximately 6–6.5 ms. A characteristic feature of the force-time curves for impacting metal-fiber laminates, as recorded during the impact process, are force fluctuations occurring particularly at the stage of strength increase [32]. The resulting f–t curves demonstrated force fluctuations, and, at the same time, we observed that their intensity varied depending on the MVF for the same impact energies. In the 5 J impact energy range, it was noticed that in laminates using 0.3 mm thick sheets (MVF = 0.375 and 0.49, laminates type A and C, respectively), there were force fluctuations, especially at the stage of force increase, while in laminates with 0.5 mm thick titanium layers (MVF = 0.4 and 0.6, laminates type B and D, respectively), no force fluctuations of comparable intensity were observed. The observed fluctuations occur in the range of force close to the P_m_ value, which may indicate the start of the laminate degradation process through the initiation of numerous matrix cracks and delaminations [33]. In laminates where no significant force fluctuations were recorded at 5 J impact energy, we can expect a more stable development of damage, probably initiating in less numerous areas, resulting from local but consistent deformation of the laminate at the point of impact [31]. In other energy ranges, i.e., 15, 30, and 45 J, similar relationships were observed, with the most intense fluctuations occurring in the same force values as in the case of 5 J of energy. This indicates the regularity of laminates in the initiation of damage propagation regardless of their thicknesses of layers factor. The analysis of the f-t curves for 30 J impact energy did not clearly reveal the construction factor effect in the qualitative assessment of the course of the force–time relationship, nor in the shape of the graphs or as the total contact time. Nevertheless, it was possible to observe that the laminate with the highest MVF value was characterized by the smallest force fluctuations in the force increase zone, similarly to what was observed in case of 5 J and 15 J impacts. In the case of impact energy of 45 J, for all laminates, there was no unequivocal impact of the laminate construction factor in the qualitative assessment of the force course over time, the shape of the charts, or as the total contact time. Finally, we discovered that the laminate with the highest MVF value has the smallest force fluctuations in the force increase zone, similar to impacts in the energy range of 5–45 J. This may be due to the greater proportion of elastic-plastic metal in such laminates, as compared to laminates with low MVF, where the proportion of matrix that is fragile and susceptible to cracking is greater [3]. The few fluctuations observed on the f–t curves for 30 and 45 J energy in the range of forces close to the maximum force may indicate the beginning of the process of fiber cracking, which, after the base damage and delamination, constitute the next stage of laminate degradation—that which occurs immediately before the initiation of cracking of metal layers and of perforation [31].

**Figure 3:**
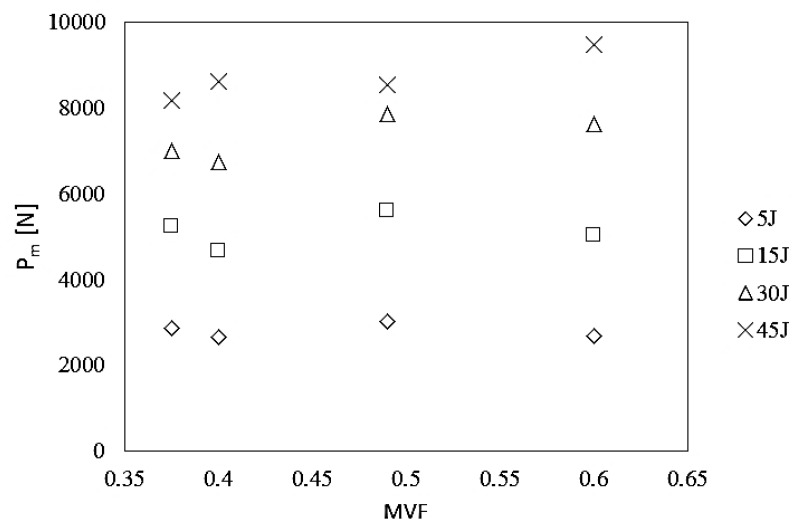
The relation between maximum force and metal volume fraction in HTCL under different impact energies.

The detailed maximum force values depending on the MVF coefficient of the HTCL laminate in the ranges of impact energy of 5 to 45 J are found in Figure 3.

Based on the analysis of the chart of the relation between maximum force and metal volume fraction in HTCL under different impact energies, we found that the maximum force values do not change significantly depending on changes in the laminate MVF. Both laminates with extreme metal volume fraction coefficients, i.e., MVF = 0.375 (HTCL type A) and MVF = 0.6 (HTCL type D), and that with intermediate MVF values, have a similar level of maximum force regardless of the impact energy. Building upon the above observations, it can be established that MVF in the range of 0.375–0.6 has no effect on the value of forces acting on the laminate during impacts with energies in the range of 5–45 J. Nakatani et al. [14] presented force–time changes for hybrid titanium glass laminates. Their results and data interpretation show a marked similarity with the presented force and time values. This is also the case for the smooth shape of force–time curves. It demonstrates that the material in the basic configuration (2/1 and 3/2) has repeatable and high impact resistance.

**Figure 4:**
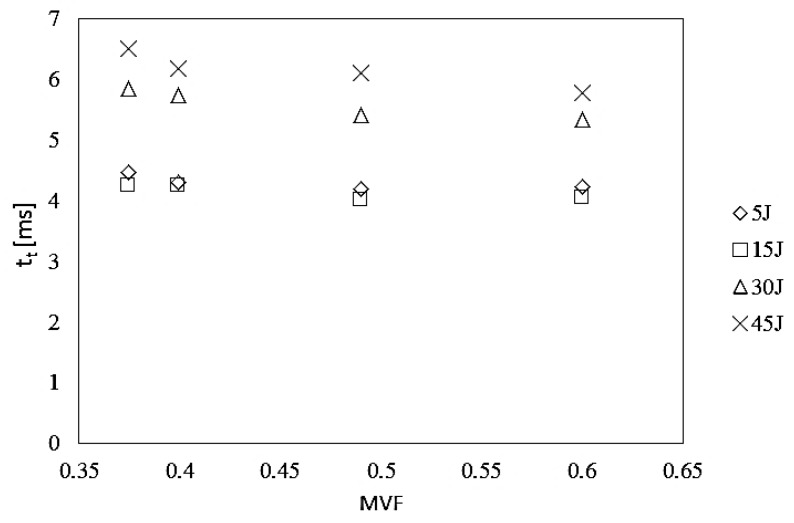
The dependence between total impact time and metal volume fraction in HTCL for different impact energies.

The experimentally recorded values of the total indenter contact time with the laminate (*t*_t_) were analyzed in detail. The recorded *t*_t_ values in juxtaposition to the MVF coefficients for various impact energies are shown in Figure 4.

Based on the analysis of the distribution of points corresponding to the values of the total indenter’s contact time with the laminate at impacts in the energy range of 5–45 J and various MVF coefficients of laminates, a tendency of shortening the total time of the indenter’s impact on the laminate with the increase of MVF was noted, especially in the area of higher impact energies, i.e., 30 and 45 J. This phenomenon may result from the greater ability of these laminates to accumulate energy (due to a greater proportion of metal in the process of energy transfer through the indenter), which results in greater capacity for faster laminate return to the elastic range after reaching its maximum deflection. The authors of other papers also reached similar conclusions about the capacity to accumulate energy demonstrated by FML laminates, including laminates based on titanium [4,9,14,32,33,34,35].

### 3.2. The Energy Absorption Process

It is possible to estimate the value of the energy absorbed (*E_a_*(*t*)) by the laminate during the process of impact [16]. Dependencies (2)–(5) are the solution of calculations of the absorbed energy based on experimental data such as displacement, velocity, and force. As it is not possible to measure absorbed energy directly, the indirect method based on physical and measurable parameters was used (see Equations (2)–(5)).

(2) $$E_{a}\left(t\right)=\frac{m\left(v_{i}^{2}-\;v^{2}\right)}{2}+mg\delta$$

where:

*δ*—indenter displacement [M];
*v_i_*—velocity of impacting body at the moment of contact [m/s];
*v*—final velocity [m/s];
*m*—mass of the indenter [kg];
*g*—gravitational acceleration, 9.80665 m/s^2.^

The form of the Equation (2) must be transformed into a form that will include the variable parameters recorded on f–t curves, i.e., force in time (*F*(*t*)) and time (*t*). The final velocity of the indenter *v* [m/s] may be described according to Equation (3):

(3) $$v=v_{i}+gt-\int_{0}^{t}\frac{F\left(t\right)}{m}dt$$

where:

*t*—total time of indenter-material contact [s];
*F*—force measured at the time of impact [N];
*m*—mass of the indenter [kg];
*g*—gravitational acceleration.

whereas indenter displacement *δ* [m] must be described via Equation (4):

(4) $$\delta =v_{i}t+\frac{gt^{2}}{2}-\int_{0}^{t}(\int_{0}^{t}\frac{F\left(t\right)}{m}dt)dt$$

where:

*t*—total time of indenter-material contact [s]
*v_i_*—velocity of impacting body at the moment of contact [m/s]
*F*—force measured at the time of impact [N]
*m*—mass of the indenter [kg]
*g*—gravitational acceleration.

By the means of appropriate substitutions and transformations, the final expression Equation (5) determines the energy absorbed by the laminate:

(5) $$E_{a}\left(t\right)=\frac{m\left[v_{i}^{2}-{\left(v_{i}+gt-\int_{0}^{t}\frac{F\left(t\right)}{m}dt\right)}^{2}\right]}{2}+mg[v_{i}t+\frac{gt^{2}}{2}-\int_{0}^{t}(\int_{0}^{t}\frac{F\left(t\right)}{m}dt)dt]$$

**Figure 5:**
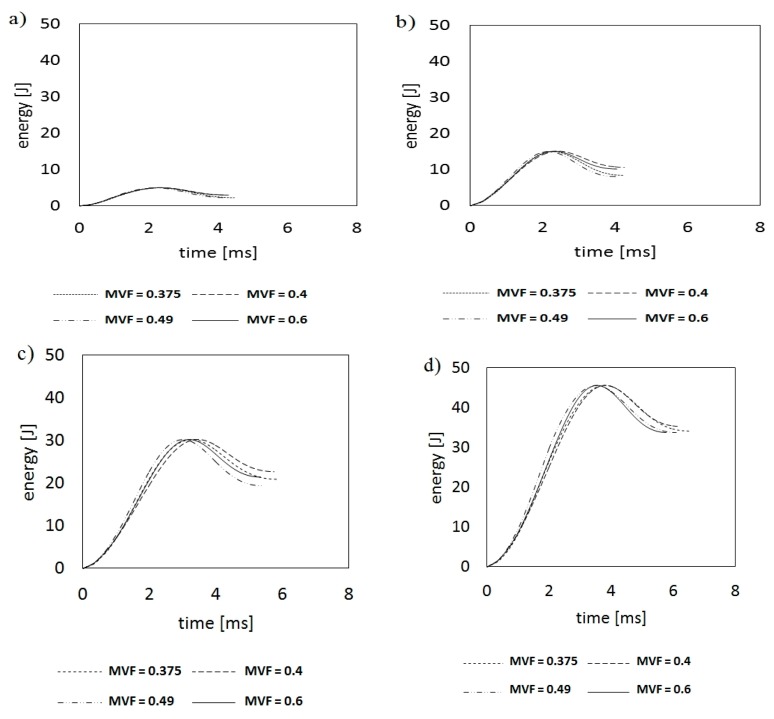
The curves represented the energy absorption process in time of HTCL with various MVF after low-velocity impact with energy 5 J (**a**); 15 J (**b**); 30 J (**c**); and 45 J (**d**).

The *E_a_*(*t*) by the laminate during the process of impact is the final energy value at the last time of the contact of the impactor with the laminate. The energy absorption process in impact time is presented in Figure 5.

The energy–time curves are characterized by the stage of energy increase to a maximum value equal to the kinetic energy of the striking body just before its contact with the laminate and the subsequent decrease in energy to a value depending on the material’s energy absorption capacity [16,23,24]. It was noticed that as the energy increases, the difference between maximum energy and absorbed energy changes. For lower impact energies, the graphs are characterized by having a flatter shape. This demonstrates a low level of energy absorption by the laminate, and that with the increase of impact energy, the level of absorbed energy increases significantly. Moreover, at the same time, it reveals greater differences in absorbed energy in relation to maximum energy. An increase in the level of energy absorbed by the laminate in the range of higher impact energy directly indicates a significantly greater share of laminate structure degradation (including even the share of local fiber cracking) that can be attributed to taking on a significant share in the energy absorption process (fiber dominant damage mechanism) [35]. This also corresponds to the conclusions stemming from the f-t curve analysis for higher impact energies (30 and 45 J) of HTCL.

**Figure 6:**
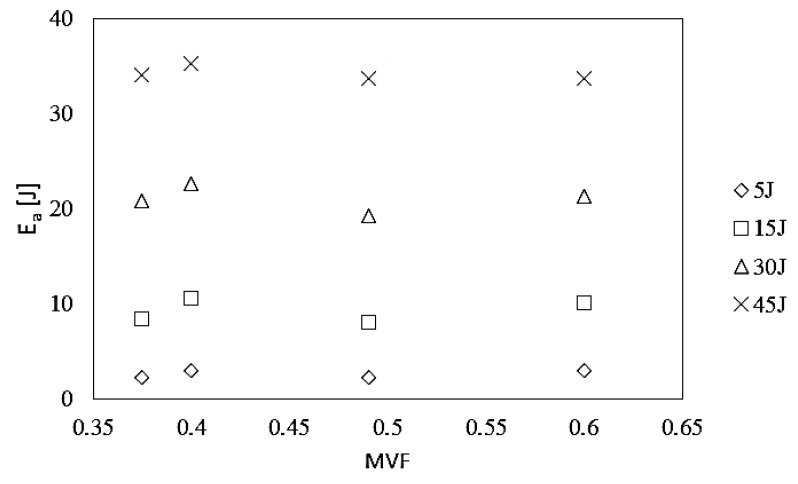
Absorbed energy vs. metal volume fraction in HTCL for various impact energies.

Figure 6 shows the relationship between the value of absorbed energy and the metal volume fraction parameter of the HTCL laminate for impact energies in the range of 5 to 45 J.

As a result of the analysis of the impact of MVF in titanium–carbon composite laminates on the value of absorbed energy, based on energy–time curves, a slight tendency can be found that laminates with a higher MVF = 0.49 and MVF = 0.6 ratios (HTCL type C and D, respectively) are more often characterized by lower values of absorbed energy than that of laminates with a lower metal content. This is due to the greater share of the brittle composite, where energy absorption is based on the increasingly dominant cracking of the polymer phase and the boundary (for lower impact energies [36] and fibers in the range of higher impact energies [32,33]. This is due to the elastic deformation of the metal and its subsequent transfer to the indenter in the reflection stage [23]. Ultimately, it can be stated that HTCL laminates with a lower total thickness of the metal part have a slightly better ability to absorb impact energy in the higher impact energy range (30 J, 45 J) due to reaching the end of the deformation capacity (by means of elastic strain) of the composite part of HTCL and starting the failure initiation and propagation of carbon fibers [31].

### 3.3. Bending Stiffness Parameter

**Figure 7:**
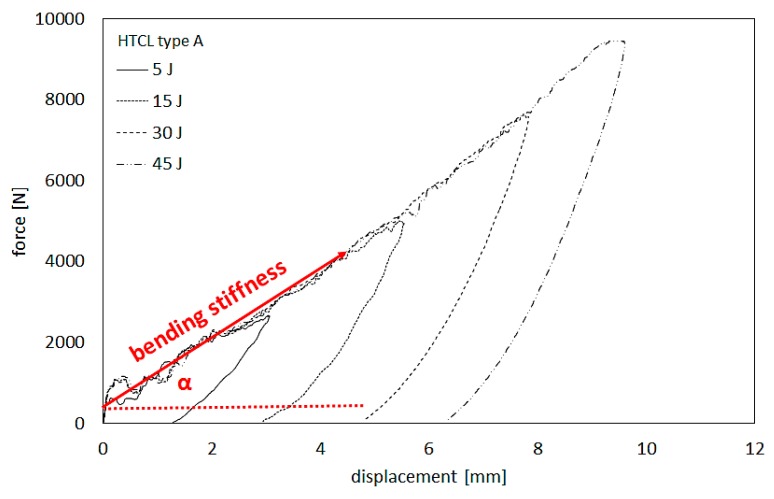
An example of force–displacement curves after impact with a visualization of bending stiffness.

Impact bending stiffness can be described as the initial bend angle of a rectilinear section of force increase as a function of displacement. Bending stiffness calculations were conducted on the basis of force-displacement curves (Figure 7) according to the data provided in the literature [37,38,39]. Figure 7 presents a visualization of the bending stiffness calculation method.

Bending stiffness was calculated as tgα, where α is the slope angle of the straight part of the increase in force as a function of displacement. The assessment employs the ascending section of the force-displacement curve following the system stabilization stage (Figure 7) [31]. Table 3 shows selected bending stiffness values of individual laminates.

Bending stiffness may be employed as one of the quantitative criteria for assessing the laminate and composite impact resistance due to its representation of the stiffness of laminates under impact-induced bending in the beginning of the impact process [36]. Based on the determined bending stiffness values, it can be observed that bending stiffness is more or less constant for a given laminate and generally independent of impact energy. The variation does not exceed 5.7%, 8.12%, 9.63%, and 8.7% for HTCL types A, B, C, and D, respectively (Table 3). A comparison of bending stiffness values between HTCL with various proportions of metal layer thickness in total laminate thickness indicates that lower bending stiffness is present in laminates with a lower proportion of metal thickness (37.5% and 40%—first group), unlike for higher total titanium thickness (49% and 60% of total laminate thickness—second group). The extreme difference of bending stiffness value in group one under the same impact energy was 11.4%, while in second group 11.7%. It can be stated that different composition (2/1, 3/2, or 4/3 lay-up configuration), different interface density, single-layer thickness, thickness ratio, and stress state in the laminate due to transverse load transfer paths are not significant features for impact response understood as bending stiffness and the deformation process of HTCL. This is probably caused by the high influence of titanium in the whole deformation and global damage process in HTCL (metal dominant failure mechanisms) under the impact process [4]. The similarity of bending stiffness of the tested HTCL is due to this material property depending mostly on its thickness of laminates [39] and the total thickness of the laminate was constant.

### 3.4. KER Coefficient

The values of kinetic energy restitution coefficient (*KER*) were calculated for the purpose of obtaining a quantitative assessment of impact behavior of HTCL with various MVF. The *KER* coefficient is used to some extent to compare the impact behavior of FMLs [40]. *KER* can be defined as a squared ratio of impactor velocity before and after impact (Equation (6))

(6) $$KER=\frac{v_{0}{}^{2}}{v_{a}{}^{2}}$$

where:

$v_{0}$—velocity of the impactor at the contact moment with HTCL surface;
$v_{a}$—velocity of the impactor at the disconnect moment with HTCL surface.

In planning *KER* analysis to compare the impact resistance of composites, the most important fact to be taken into account is the observation of perforations in each sample. Because of the possibility of total perforation of composites or FML, impactor velocity can be similar when the impactor is rebounded or goes through to the other side of the laminate. However, the impact behavior of the material is extremely different in the above phenomena. This is the reason that completely perforated materials (passage of the impactor through the laminate to the opposite side) cannot be compared by *KER* coefficient with laminates that are not completely perforated. Table 4 presents the values of the calculated *KER* coefficient for tested HTCLs.

The kinetic energy restitution coefficient shows that the tested laminates with various metal content could be divided into two groups. The first one, which contains HTCL types A and B, is characterized by 25%, 30%, 19%, and 6.4% (5, 15, 30, and 45 J impact energies, respectively) and lower *KER* (on average) in comparison to HTCL types C and D (second group). The differences in *KER* parameters between the abovementioned groups are more pronounced under lower-impact energies because during low-energy impact, the elastic deformation of FML laminates serves as the main energy absorption mechanism. This suggests that HTCLs with lower total thickness of metal layers have a greater ability to accumulate energy. This is probably due to the elastic nature of strain in fibrous composites, which make up the majority of the volume in such laminates. The important fact is that after higher impact energy, the elastic strain of the composite part of FML comes to an end, giving way to matrix crushing and fiber breaking as the primary mechanisms of energy absorption in the composite part [3,31,35]. The calculated *KER* parameters correspond with the energy absorption capacity of HTCL laminates with various MVF coefficients as well as with bending stiffness parameters (lower bending stiffness for lower metal content). It can be stated that *KER* parameters are higher than the kinetic energy of the impactor after the impact process, which indicates that HTCLs with MVFs = 0.375 and 0.4 have a lower ability to absorb energy, but these phenomena are more pronounced in the low impact energy range (up to 5 J) [36].

### 3.5. The Final Damage Appearance

**Figure 8:**
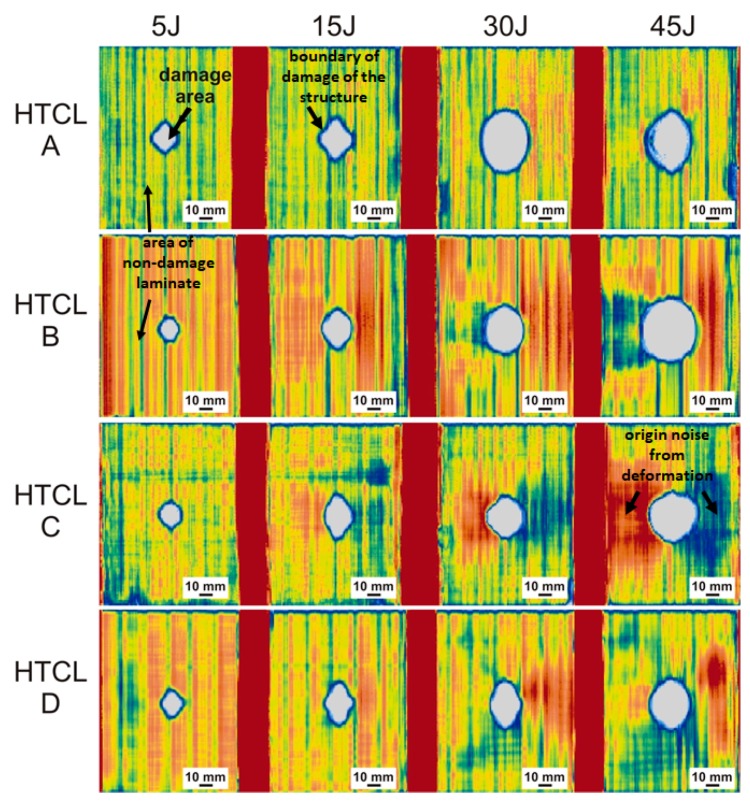
C-scan maps of damage of HTCL with various metal volume fractions after impact.

The C-scan maps of laminates and their damage after impact are presented in Figure 8.

According to the applied NDT method presented in detail in papers by other authors [29], the white areas in C-scan maps are placed exactly where laminate internal structure damage (matrix cracks and delamination) came about. The central white area is the area with delamination at the metal-composite or composite-composite interface [29]. The blue ring around the damaged area is the boundary of the damage area where a diffraction of the ultrasonic wave changes the C-scan color (attenuation). The red, yellow, and blue area is the non-damaged FML structure. Different colors represent different attenuation levels because of the plastic deformation of samples (locally differentiated wave reflection). However, as it was proved [29], the signal between the transmitting and receiving transducers means that there is no damage to material continuity. Based on the analysis of C-scan images (view from the surface), all samples tested revealed damage in the central part of the plate, near the point of impact. Furthermore, qualitative, visual analysis of ultrasonic C-scan maps demonstrated that in all cases subjected to analysis, the total damaged area increased with the increase of impact energy. Shapes similar to a circle or oval characterized a significant part of the damaged areas. However, in the case of a laminate with MVF = 0.6, after impact with energies of 5 and 15 J and the MVF = 0.375 laminate subjected to 15 J impact energy, an irregular shape of the surface of the destruction was observed, forced by the orientation of fiber arrangement in the laminate. The aforesaid results from the dominant role of propagation of delamination at the border of layers with different fiber orientation in the total damage [16].

Based on the analysis of Table 5, we noticed that as the energy of the impact increases, the surface area of laminate destruction increases, regardless of the specific laminate structure. HTCL laminates with a lower metal volume, in general, are characterized by higher proportion of surface degraded. This comes about as the result of loading with the same energy, when compared to laminates with a higher MVF. The observed phenomenon can be explained by greater share of the composite, in particular, the brittle matrix, in energy absorption by crack propagation. Type D HTCL laminate, which had the highest MVF, demonstrated the relatively least extensive destruction of the internal structure. Referring to the analysis of energy absorption curves during impact, it can be concluded that the presence of a significant amount of metal in the laminate volume limits the propagation of matrix cracks and delamination. It is important that the discussed differences in the amount of damage to laminates with MVF in the range of 0.375–0.6 are not significant for individual impact energies. If it is considered that titanium/composite interface fracture toughness is much lower in comparison to aluminum/composite, the meaning of interfaces number probably is strongly limited (in the range of 2 to 6—such minimum and maximum numbers of metal/composite interfaces were considered). This is perhaps the reason that the true results do not show sensitivity on impact behavior because of increasing the interfaces in the mentioned range, even if some authors shows dependences for GLARE laminates [25]. Because of that, the number of interfaces was not a reference point in the manuscript. The maximum differences in share of damage in the total area of the laminate are 0.6%, 0.44%, 4.64%, and 2.84% for strokes with energy of 5, 15, 30, and 45 J, respectively. At the same time, it can be seen that the damage analysis of HTCL with various metal content and number of interfaces correlates with the observations of curves representing energy changes over time, where a greater ability to absorb impact energy of HTCL with a lower MVF coefficient was recorded, precisely due to the increased share of composite destruction, especially at the metal composite phase boundary and/or the boundaries of composite layers arranged perpendicularly to each other [16,41].

## 4. Summary

The objective of the present work was to evaluate the influence of metal volume fractions to low-velocity impact resistance. This assessment was built upon the example of innovative hybrid titanium–carbon composite laminates. This study was based on experimental research, numerous evaluation criteria, and comparison of impact resistance. The quantitative and qualitative impact behavior analyses for laminates with various thicknesses and numbers of metal and composite layers (laminate composition described by metal volume fraction coefficient in the range from 0.375 to 0.6), enabled the authors to formulate the following observations:

- The laminate with the highest total thickness of the metal part has the smallest force fluctuations in the force increase zone, similar to impacts in the energy range of 5–45 J. This may be due to the greater proportion of elastic-plastic metal in such laminates, as compared to laminates with higher content of composite layers, where the proportion of matrix that is brittle and susceptible to cracking is greater.
- In the qualitative analysis of the influence of metal and composite layer thicknesses in titanium–carbon composite laminates on the value of absorbed energy, based on energy–time curves, a slight tendency can be found according to which laminates with higher total thickness of titanium layers (the proportion of 49% and 60% of total titanium thickness in total laminate thickness) are more often characterized by a lower value of absorbed energy than laminates with a lower metal content. This may be due to the greater proportion of brittle composite in the laminates wherein the energy absorption relates to the greater proportion of brittle composite cracking (the total thickness of carbon/epoxy layers is 60% or more of the total thickness of laminate). However, the changes recorded are insignificant and cannot prove the advantage of laminates with a specific MVF in terms of impact resistance.
- A tendency to shorten the contact time of the indenter with the laminate was observed, regardless of the impact energy. This phenomenon may result from the greater ability of these laminates to accumulate energy (due to a greater proportion of metal in the process of energy transfer through the indenter), which results in greater capacity for faster laminate return to the elastic range after reaching its maximum deflection.
- HTCL laminates with a lower metal content, where the total thickness of metal layers is lower than total composite thickness, are generally characterized by a greater size of degradation as a result of loading with the same energy when compared to laminates with a higher contribution of metal layers in the damage process. The observed phenomenon can be explained by the greater share of the composite, including, in particular, the brittle matrix, in energy absorption by crack propagation, and the expansion of delaminations in brittle carbon composite, especially after impact with a relatively higher impact energy.
- Hybrid titanium/carbon-epoxy laminates, characterized by constant total thickness, but with various thicknesses of individual layers, loaded by low-velocity impact demonstrate that increasing the total thickness of the metal part, even if the metal part is divided into a higher number of layers, contributes to a higher energy absorption capacity due to starting the domination of metal dominant failure mode. At the same time, increasing the density of the interfaces does not strongly affect the damaged area, the limits of impact forces, bending stiffness, and kinetic energy restitution. The above phenomena were observed in the range of metal volume fraction from 0.375 to 0.6, in hybrid titanium/carbon laminates with lay-up schemes of 2/1; 3/2; and 4/3.

On the basis of the conducted research and analyses, it can be finally stated that no significant differences in maximum force values, total contact time, and damage range were found. However, some tendency to limitation of the energy accumulation capacity with an increase in metal volume fraction was observed, especially in the lower-impact energy range. This can result in a lower bending stiffness of laminates with lower metal volume fraction and possibility of the elastic strain of the composite part before the initiation of the damage process of fibers. Finally, as some features of laminates, such as different interface density, single-layer thickness, thickness ratio, or the stress state in laminate due to the transverse load transfer paths were not considered, it can be stated that the mentioned features have limited influence on the global impact response of HTCLs with various metal volume fractions.

## Figures and Tables

**Table 1:** HTCL composite laminates details.

Sample Name	Lay-Up Scheme	Number of Titanium Layers	Number of Single Carbon-Epoxy Layers	Titanium Layer Thickness (*t*_m_) [mm]	Total Laminate Thickness (*t*_lam_) [mm]	Metal Volume Fraction (MVF)
A	3/2	3	12	0.3	2.5	0.375
B	2/1	2	12	0.5	2.5	0.4
C	4/3	4	10	0.3	2.5	0.49
D	3/2	3	8	0.5	2.5	0.6

**Table 2:** Parameters of impact experiments.

Impact Energy [J]	Impactor Mass [kg]	Impactor Velocity * [m/s]	Impactor Height [mm]
5	2.006	2.23	253.5
15	4.006	2.74	382
30	4.006	3.87	763.6
45	4.006	4.74	1145

* exactly at the moment of contact with the sample.

**Table 3:** Bending stiffness of tested HTCL with various layer thicknesses.

Proportion of Metal Layer Thickness in Total Laminate Thickness [%]	Bending Stiffness [N/mm]			
	Impact Energy			
	5 J	15 J	30 J	45 J
37.5 HTCL type A	870	867	919	889
40 HTCL type B	849	805	814	825
49 HTCL type C	1205	1098	1166	1115
60 HTCL type D	1080	1038	1029	996

**Table 4:** The kinetic energy restitutionz (*KER)* coefficient of HTCLs with various MVF coefficients.

Impact Energy [J]	5	15	30	45
	*KER*			
HTCL type A MVF 0.375	2.49	3.07	3.44	3.97
HTCL type B MVF 0.4	2.48	3.39	4.02	4.45
HTCL type C MVF 0.49	1.85	2.16	2.79	3.88
HTCL type D MVF 0.6	1.84	2.27	3.25	4.00

**Table 5:** Damage area of HTCL with various MVF after impact.

Laminate Type	Damage Size	Impact Energy [J]			
		5 J	15 J	30 J	45 J
HTCL A MVF = 0.375	D_a_ *	249 mm^2^ 1.66%	436 mm^2^ 2.91%	1191 mm^2^ 7.94%	854 mm^2^ 5.69%
	(D_a_/T_a_)*100% **				
HTCL B MVF = 0.4	D_a_	159 mm^2^ 1.06%	388 mm^2^ 2.59%	673 mm^2^ 4.49%	1279 mm^2^ 8.53%
	(D_a_/T_a_)*100%				
HTCL C MVF = 0.49	D_a_	182 mm^2^ 1.22%	386 mm^2^ 2.57%	495 mm^2^ 3.3%	995 mm^2^ 6.64%
	(D_a_/T_a_)*100%				
HTCL D MVF = 0.6	D_a_	180 mm^2^ 1.2%	366 mm^2^ 2.44%	525 mm^2^ 3.5%	995 mm^2^ 6.64%
	(D_a_/T_a_)*100%				

* D_a_-damage area [mm^2^]; ** (*D*_a_/*T*_a_)*100%—share of damage in the total area (*T*_a_) of the laminate [%].
